# Clinical features of subclinical left ventricular systolic dysfunction in patients with diabetes mellitus

**DOI:** 10.1186/s12933-015-0201-8

**Published:** 2015-04-17

**Authors:** Yasuhide Mochizuki, Hidekazu Tanaka, Kensuke Matsumoto, Hiroyuki Sano, Hiromi Toki, Hiroyuki Shimoura, Junichi Ooka, Takuma Sawa, Yoshiki Motoji, Keiko Ryo, Yushi Hirota, Wataru Ogawa, Ken-ichi Hirata

**Affiliations:** Division of Cardiovascular Medicine, Department of Internal Medicine, Kobe University Graduate School of Medicine, 7-5-2, Kusunoki-cho, Chuo-ku Kobe, 650-0017 Japan; Division of Diabetes and Endocrinology, Department of Internal Medicine, Kobe University Graduate School of Medicine, Kobe, Japan

**Keywords:** Diabetes mellitus, Nephropathy, Albuminuria, Echocardiography, Two-dimensional speckle-tracking strain, Global longitudinal strain

## Abstract

**Background:**

Left ventricular (LV) longitudinal systolic dysfunction has been identified even in asymptomatic patients with diabetes mellitus (DM) and preserved LV ejection fraction (LVEF). However, its relevant clinical features have not been fully evaluated.

**Methods:**

We studied 144 asymptomatic DM patients without coronary artery disease. Their mean age was 57 ± 15 years, 79 (55%) were female, and mean LVEF was 66 ± 4% (all ≥50%). Global longitudinal strain (GLS) was determined as the average peak strain of 18 segments from the three standard apical views, and was expressed as an absolute value. With the pre-defined cutoff for subclinical LV systolic dysfunction in DM patients with preserved LVEF set at GLS < 18%, this dysfunction was detected in 53 patients (37%).

**Results:**

Multivariate logistic regression analysis revealed that type 2 DM, hypertriglyceridemia, overweight/obesity, nephropathy and neuropathy were independently associated with GLS < 18%, with nephropathy being the highest risk factor (OR: 5.26; 95% CI 2.111-13.12, p < 0.001). For sequential logistic regression models, a model based on clinical variables including gender, type 2 DM and DM duration (*χ*^2^ = 24.1) was improved by addition of overweight/obesity and hypertriglyceridemia (*χ*^2^ = 45.6, p < 0.001), and further improved by addition of nephropathy and neuropathy (*χ*^2^ = 70.2, p < 0.001) as variables. Furthermore, albuminuria significantly correlated with GLS (r = −0.51, p < 0.001), and a multivariate regression model showed it to be the factor most closely associated with GLS (β = −0.33, p < 0.001).

**Conclusions:**

Diabetic complications, hypertriglyceridemia and overweight/obesity were closely associated with early stage of LV systolic longitudinal myocardial dysfunction in asymptomatic DM patients with preserved LVEF. Our findings can be clinically noticeable for the management of DM patients.

## Background

Diabetes mellitus (DM) is considered a major contributor of the development of heart failure (HF) despite absence of coronary artery disease and hypertension even in patients with preserved left ventricular (LV) ejection fraction (EF). This condition is known as diabetic cardiomyopathy [1-3]. Although the pathogenesis of diabetic cardiomyopathy is believed to be multifactorial but with the exact cause remaining unknown, a number of mechanisms such as hyperglycemia and hyperinsulinemia have been reported to play an important role in its etiology. These changes are observed as changes in free acid metabolism, increased apoptosis, activation of the renin-angiotensin system, abnormalities in copper metabolism, autonomic neuropathy, stem cell defect, and increased oxidative stress among others. All these underlying pathogenetic conditions change the cardiac structure and may lead to cardiac fibrosis [1,4]. Diabetic cardiomyopathy is currently defined as a diastolic dysfunction, and several studies of DM patients have identified LV diastolic dysfunction as the earliest functional alteration in the course of diabetic cardiomyopathy [5-9], and also established it as an important prognostic parameter [6]. On the other hand, LV longitudinal myocardial systolic dysfunction has been identified in DM patients with preserved LVEF without overt coronary artery disease or HF [10-16]. In addition, recent investigations have found that LV longitudinal myocardial systolic dysfunction, rather than LV diastolic dysfunction, should be considered the first marker of a preclinical form of diabetic cardiomyopathy in DM patients with preserved LVEF without overt HF [14,17]. However, which characteristics of DM patients are associated with impaired LV systolic longitudinal myocardial function is not fully understood. Accordingly, our objective was to evaluate the factors associated with the clinical features of impaired LV longitudinal myocardial systolic function in asymptomatic DM patients with preserved LVEF.

## Methods

### Study populations

A series of 150 consecutive DM patients including type 1 and type 2 DM who were admitted to Kobe University Hospital between July 2013 and November 2014 were prospectively recruited for this study. The diagnosis of DM and its type were established according to the World Health Organization criteria [18]. We excluded patients with (1) ischemic heart disease; (2) LVEF < 50%; (3) a previous history of open-heart surgery; (4) severe types of renal dysfunction defined as glomerular filtration rate (GFR) <30 mL/min/1.73 m^2^; (5) hypertension >180/100 mmHg uncontrollable despite medical therapy; (6) significant valvular heart disease; (7) atrial fibrillation; and (8) left or right bundle branch block. All patients underwent exercise stress testing such as treadmill exercise or stress myocardial perfusion scintigraphy within at least 2 weeks after admission, and none of the patients showed an ischemic response. Six initially eligible patients (4%) were excluded from all subsequent analyses because of suboptimal images from poor echocardiographic windows. As a result, the final study group consisted of 144 patients. The study protocol was approved by the ethics committee of our institution and all patients gave informed consent before participation.

### Echocardiographic examination

All echocardiographic studies were performed using a commercially available echocardiographic system within at least 2 weeks after admission (Vivid E9; GE-Vingmed, Horten, Norway). Digital routine grayscale two-dimensional cine loops from three consecutive heartbeats were obtained at end-expiratory apnea from the standard parasternal long-axis view and three apical views at depths of 12–14 cm and mean frame rates of 67 ± 8 frames/sec. Sector width was optimized to allow for complete myocardial visualization while maximizing the frame rate. Digital data were transferred to dedicated software (EchoPAC version113; GE Vingmed) for subsequent offline analysis. Standard LV measurements were obtained in accordance with the current guidelines of the American Society of Echocardiography/European Association of Cardiovascular Imaging [19]. LV volumes and LVEF were calculated using the modified biplane Simpson’s method, which was also employed to calculate left atrial volume using apical 2- and 4-chamber views at the ventricular end-systole, and then normalized to body surface area. LV mass was indexed to both body surface area and height^2.7^, considering influence of obesity. LV stroke volume was determined in terms of the velocity-time integral and assessed by means of pulsed-wave Doppler positioned at the LV outflow tract. The early diastolic (E) and atrial wave velocities and the E-wave deceleration time were measured using pulsed-wave Doppler recording from the apical four-chamber view. Spectral pulsed-wave Doppler-derived early diastolic velocity (E’) was obtained from the septal mitral annulus, and the E/E’ ratio was calculated to obtain an estimate of LV filling pressure [20].

### Assessment of GLS

**Figure 1:**
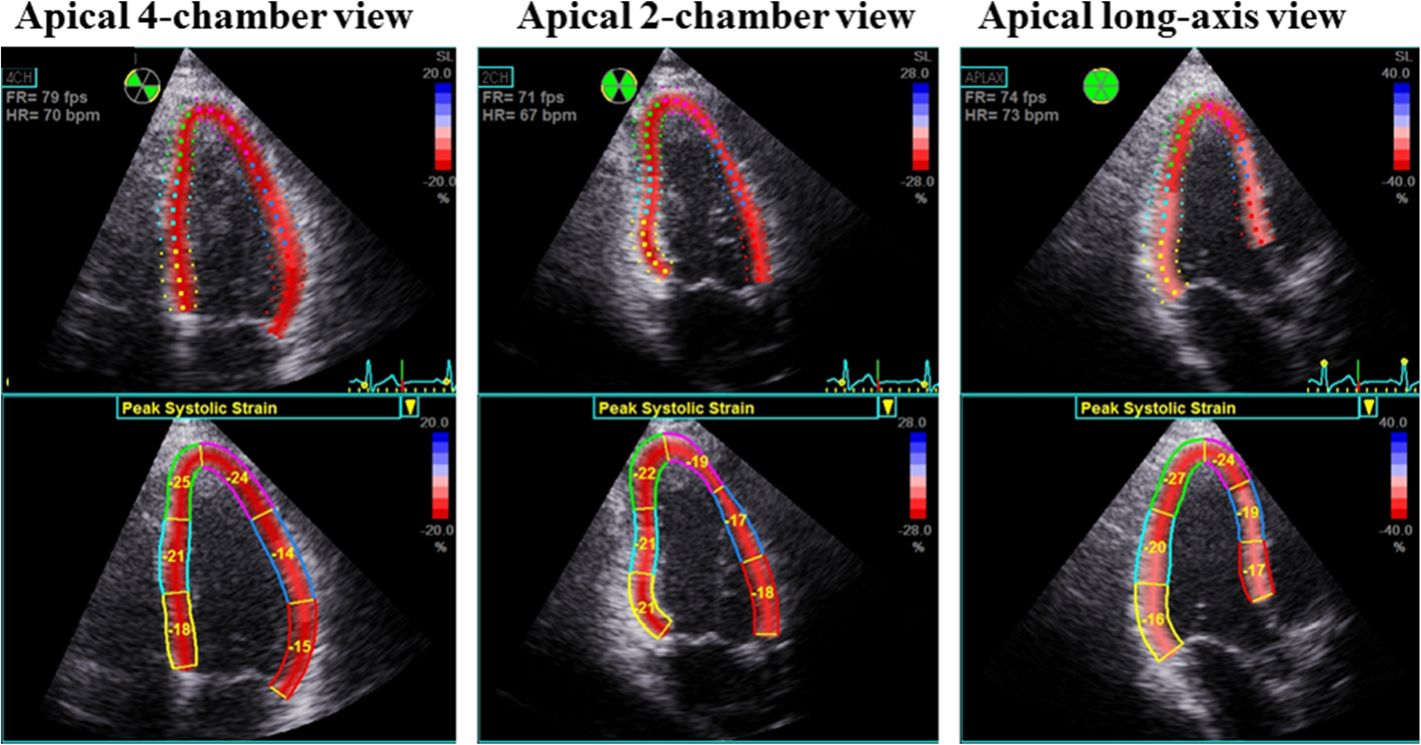
Example of color-coded 2-dimensional left ventricular (LV) display derived from the three standard apical views and corresponding peak longitudinal strain values derived from 18 LV segments for measurement of global longitudinal strain (GLS). GLS was determined as the average peak strain of the 18 LV segments, and was expressed as an absolute value.

Speckle-tracking strain analysis was performed for each patient with the aid of a single dedicated software (EchoPAC version 113; GE Vingmed). GLS was assessed by means of two-dimensional speckle-tracking strain from the three standard apical views as previously described in detail. Briefly, a region of interest was traced on the endocardium at end-systole with a point-and-click approach for each of the three apical views. A second larger region of interest was then generated and manually adjusted near the epicardium. Apical images were divided into six standard segments and six corresponding time-strain curves were generated. GLS was determined as the averaged peak strain of 18 segments from the three standard apical views [19], and was expressed as an absolute value (Figure 1). As previously detailed, the pre-defined cutoff for subclinical LV systolic dysfunction in DM patients with preserved LVEF was set at GLS < 18% [13-15,21].

### Assessment of DM-related clinical features

Overweight/Obesity was defined as body mass index (BMI) ≥25 kg/m^2^ according to World Health Organization’s definition. Hypertension was defined as systolic blood pressure ≥ 140 mmHg or diastolic blood pressure ≥ 90 mmHg, or currently use of medication for hypertension [22]. Dyslipidemia was defined as fasting low-density lipoprotein ≥ 140 mg/dl, or currently use of medication for dyslipidemia [23]. Similarly, hypertriglyceridemia was separately defined as fasting triglyceride ≥ 150 mg/dl, or currently use of medication for hypertriglyceridemia [23]. Fasting hemoglobin A1c, 1,5-anhydroglucitol, glycoalbumin, creatinine, estimated glomerular filtration rate and lipid profile were obtained on the day after admission. Albuminuria was determined on the basis of the average measurement of albumin in urine collected over three 24-hour periods. Nephropathy was defined as albuminuria at least ≥30 mg/day (but GFR > 30 ml/min/1.73 m^2^) [24]. Experienced diabetologists assessed the presence of diabetic neuropathy according to current guidelines and with reference to a nerve conduction study [25]. Moreover, diabetic retinopathy was defined when patients matched with any one of following graduated classifications: microaneurysms only, mild-moderate, or severe non-proliferative diabetic retinopathy, and proliferative diabetic retinopathy or prior retinal photocoagulation by the experienced ophthalmologists [26].

### Statistical analysis

Continuous variables were expressed as mean values and standard deviation for normally distributed data and median and interquartile range for non-normally distributed data, while categorical variables were expressed as frequencies and percentages. The parameters of the two subgroups were compared by using Student *t* test or Mann- Whitney *U* test as appropriate. Proportional differences were evaluated with Fisher’s exact test. Relationships between two variables were analyzed by means of linear regression and were expressed as Pearson correlation coefficients. The associations of baseline clinical parameters with reduced GLS were identified by logistic regression in univariate and multivariate analyses. Variables with p-values <0.10 were incorporated into the multivariate model by means of stepwise selection. There was not multicollinearity between variables in the multivariate model. A sequential logistic model for GLS < 18% was created to determine the incremental enhancement of the prognostic value of the patient’s characteristics including obesity, nephropathy, and neuropathy compared to that of clinical variables including age, gender and DM duration. A statistically significant increase in the global log-likelihood *χ*2 of the model was used to determine the incremental enhancement of the prognostic value. The confounding factors for logistic regression analysis and sequential logistic regression models were based on the associated factors with subclinical LV dysfunctions in DM patients which were previously reported. The inter-observer and intra-observer variability of GLS was expressed as the absolute difference between the measurements divided by their mean value from 20 randomly selected patients. Albuminuria was converted into a logarithmic scale according to its distribution for each analysis. For all steps, a p value of < 0.05 was regarded as statistically significant. All analyses were performed with SPSS version 16.0 (SPSS, Inc., Chicago, IL) and MedCalc version 14.10.2 (MedCalc Software, Mariakerke, Belgium).

## Results

### Baseline characteristics

The baseline clinical and echocardiographic characteristics of the 144 DM patients are summarized in Table 1. Their mean age was 57 ± 15 years, LVEF was 66 ± 4%, and 79 patients (55%) were female. The intra-observer variability was 3.8% and the inter-observer variability was 4.2% for GLS.

**Table 1:** **Clinical, hemodynamic, and echocardiographic characteristics of patients**

	**All patients (n = 144)**	**Patients with GLS ≥ 18% (n = 91)**	**Patients with GLS < 18% (n = 53)**	**p value**
*Clinical Data*				
Age, years	57 ± 15	57 ± 15	57 ± 15	0.75
Female, n (%)	79(55)	55(60)	24(45)	0.09
Height, m	1.6 ± 1.0	1.6 ± 0.9	1.6 ± 1.0	0.20
Weight, kg	64 ± 15	60 ± 12	72 ± 18	<0.001
Body Mass Index, kg/m^2^	24.7 ± 4.9	23 ± 3.9	27 ± 6.0	<0.001
Systolic blood pressure, mmHg	124 ± 19	122 ± 17	130 ± 21	0.007
Diastolic blood pressure, mmHg	73 ± 11	71 ± 11	76 ± 10	0.002
Pulse pressure, mmHg	53 ± 15	51 ± 14	55 ± 17	0.03
Heart rate, bpm	69 ± 11	67 ± 11	71 ± 10	0.04
Rate Pressure Product, bpm*mmHg	9173 ± 1992	8741 ± 1812	9905 ± 2052	<0.001
Obesity, n (%)	58(40)	25(27)	33(62)	<0.001
Type 2 DM, n (%)	104(72)	55(60)	49(92)	<0.001
DM duration, years	11.8 ± 9.6	10.4 ± 8.4	13.6 ± 10.1	0.04
Hypertension, n (%)	69(48)	39(43)	30(57)	0.12
Dyslipidemia, n (%)	89(62)	53(58)	36(68)	0.29
Hypertriglyceridemia, n (%)	44(31)	17(19)	27(51)	<0.001
Smoking, n (%)	31(22)	29(32)	25(47)	0.53
*Complications*				
Nephropathy, n (%)	54(38)	13(14)	34(64)	<0.001
Neuropathy, n (%)	47(33)	19(21)	29(55)	<0.001
Retinopathy, n (%)	48(33)	29(32)	25(47)	0.07
*Biochemistry and Urinary Examination*				
HbA1c, %	8.2(7.0-9.6)	8.0(6.8-9.0)	8.7(7.3-10.0)	0.21
1,5-anhydroglucitol, g/dl	4.3(2.4-8.1)	5.1(2.7-8.6)	3.3(2.0-7.1)	0.07
Glycoalbumin, %	21.8(17.4-27.2)	21.8(18.0-28.4)	22.0(16.8-26.8)	0.52
HOMA index	1.9(1.1-4.1)	1.5(1.0-3.0)	2.5(1.3-4.8)	0.049
Low-density lipoprotein, mg/dl	103 ± 35	100 ± 35	107 ± 34	0.22
High-density lipoprotein, mg/dl	53 ± 17	54 ± 16	50 ± 17	0.23
Triglyceride, mg/dl	112(78–159)	96(66–132)	154(113–203)	<0.001
eGFR, ml/min/1.73 m^2^	77(61–89)	77(64–89)	74(58–90)	0.26
Albuminuria, mg/day	11.5(4.5-33.0)	7.0(3.0-18.0)	39.0(11.0-181.3)	<0.001
*Medications*				
Calcium channel blocker, n (%)	38(26)	21(23)	17(32)	0.25
ACEI/ARB, n (%)	58(40)	30(33)	28(53)	0.02
β-blocker, n (%)	13(9)	6(7)	7(13)	0.23
Diuretics, n (%)	10(7)	4(4)	6(11)	0.17
Statin, n (%)	68(47)	36(40)	32(60)	0.02
Insulin, n (%)	90(63)	58(64)	32(60)	0.72
DPP-4I, n (%)	55(38)	30(33)	25(47)	0.11
GLP-1RA, n (%)	13(9)	7(7)	6(11)	0.55
Sulfonylurea, n (%)	26(18)	15(16)	11(21)	051
α-GI, n (%)	26(18)	15(16)	11(21)	0.51
Thiazolidine, n (%)	13(9)	6(6)	7(13)	0.23
Metformin, n (%)	59(41)	31(34)	28(53)	0.04
*Echocardiography*				
Relative wall thickness	0.45(0.39-0.51)	0.41(0.36-0.47)	0.49(0.47-0.60)	<0.001
Left atrial volume index, ml/m^2^	28(22–34)	27(22–33)	31(23–37)	0.07
LV mass index, g/m^2^	75(63–86)	68(57–80)	83(74–94)	<0.001
LV mass index, g/m^2.7^	34(27–41)	30(25–37)	41(33–46)	<0.001
End-systolic volume, ml	26 ± 10	24 ± 9	29 ± 12	0.004
End-diastolic volume, ml	75 ± 22	71 ± 21	80 ± 23	0.004
LV ejection fraction, %	66 ± 4	67 ± 4	65 ± 5	0.002
Stroke volume, ml	62(56–72)	62(56–73)	63(56–69)	0.68
E/A	0.83(0.68-1.1)	0.86(0.7-1.2)	0.77(0.66-0.95)	0.03
E-wave deceleration time	189(163–227)	190(158–225)	187(167–235)	0.54
E’	6.3(5.0-7.5)	6.5(5.6-8.1)	5.6(4.4-6.7)	<0.001
E/E’	9.6(8.0-11.6)	9.1(7.7-11.3)	10.3(9.1-13.5)	0.002
Global longitudinal strain, %	18.8 ± 2.7	20.4 ± 1.6	16.1 ± 1.7	<0.001

Values are mean ± SD for normally distributed data and median and interquartile range for non-normally distributed data, or n (%).

DM = diabetes mellitus; HOMA = homeostatic model assessment; eGFR = estimated glomerular filtration rate; ACEI = angiotensin-converting enzyme inhibitor; ARB = angiotensin II receptor blocker; DPP-4I = dipeptidyl peptidase-4 inhibitor; GLP-1RA = glucagon like peptide-1receptor agonist; α-GI = α-glucosidase inhibitor; LV = left ventricular; E = peak early diastolic mitral flow velocity; A = peak late diastolic mitral flow velocity; E’ = Spectral pulsed-wave Doppler–derived early diastolic velocity from the septal mitral annulus.

### Comparison of baseline characteristics of patients with GLS < 18% and ≥18%

Subclinical LV systolic dysfunction, defined as GLS < 18%, was observed in 53 patients (37%), and the remaining 91 patients (63%) were classified as having preserved LV systolic function (Table 1). A comparison of the characteristics of patients with GLS < 18% and those of patients with GLS ≥ 18% showed that BMI, DM duration, fasting triglyceride, albuminuria, systolic and diastolic blood pressures, and rate pressure product were significantly larger than for the latter group, whereas HbA1c, 1,5-anhydroglucitol, and glycoalbumin, which indicates the degree of blood sugar control in the recent phase, were similar for both groups. In addition, prevalence of type 2 DM, overweight/obesity, nephropathy, neuropathy, and prescription of angiotensin-converting enzyme inhibitors or angiotensin II receptor blockers, statin and metformin for patients with GLS < 18% were significantly higher than for those with GLS ≥ 18%.

### Comparison of echocardiographic parameters for patients with GLS < 18% and ≥18%

The relative wall thickness, LV mass index, and LV volumes of patients with GLS < 18% were significantly larger than those of patients with GLS ≥ 18%. In addition, LVEF of patients with GLS < 18% was significantly lower, and E/E’ for patients with GLS < 18% was significantly higher than those of patients with GLS ≥ 18%. Finally, the left atrial volume index (LAVI) for patients with GLS < 18% tended to be larger, but the difference was not statistically significant.

### Predictors of reduced GLS for DM patients

**Figure 2:**
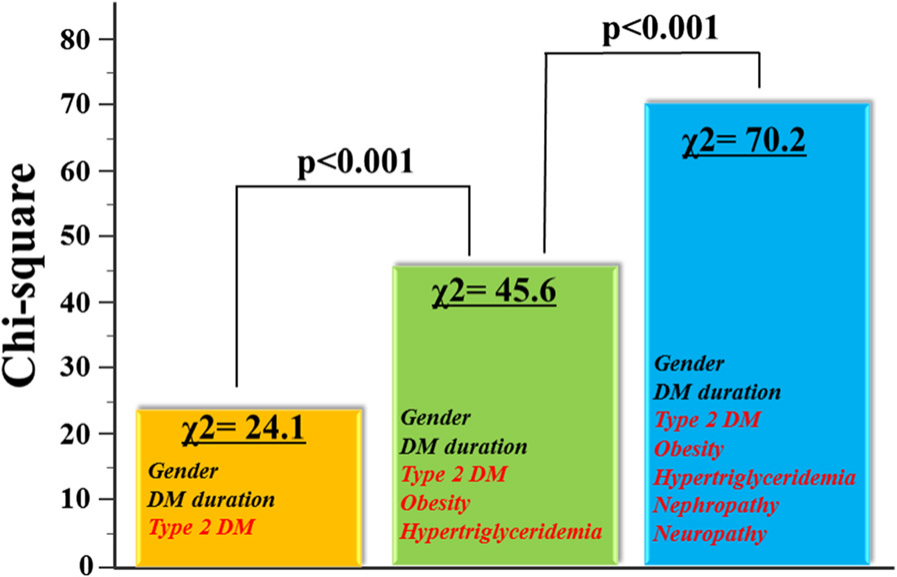
The incremental advantage of using sequential logistic models for the prediction of GLS < 18%. A model based on clinical variables including gender, type 2 diabetes mellitus (DM) and DM duration (*χ* ^2^ = 24.1) was improved by the addition of hypertriglyceridemia and overweight /obesity (*χ* ^2^ = 45.6; p < 0.001), and further improved by the addition of nephropathy and neuropathy (*χ* ^2^ = 70.2; p < 0.001).

Univariate analysis using the logistic regression model showed that type 2 DM and presence of overweight/obesity, hypertriglyceridemia, nephropathy, neuropathy, and retinopathy were associated with GLS < 18%. The odds ratio (OR) and 95% confidence interval (CI) for each of these variables are given in Table 2. An important finding of the multivariate logistic regression analysis was that type 2 DM, overweight/obesity, hypertriglyceridemia, nephropathy and neuropathy were independently associated with GLS < 18%, and nephropathy was found to be the highest risk factor of GLS < 18% (OR: 5.26; 95% CI 2.111-13.12; p < 0.001). The incremental advantage of using sequential logistic regression models for the prediction of GLS < 18% is shown in Figure 2. A model based on clinical variables including, gender, type 2 DM and DM duration (*χ*^2^ = 24.1) was improved by addition of overweight/obesity and hypertriglyceridemia (*χ*^2^ = 45.6, p < 0.001) and further improved by addition of nephropathy and neuropathy (*χ*^2^ = 70.2, p < 0.001).

**Table 2:** **Univariate and multivariate logistic regression analysis for detecting GLS < 18%**

	**Univariate**			**Multivariate**		
**Dependent variables**	**OR**	**95% CI**	**p value**	**OR**	**95% CI**	**p value**
Age	0.99	0.974-1.019	0.75			
DM duration	1.03	0.996-1.069	0.08			
Gender (female)	0.54	0.273-1.075	0.08			
Type 2 DM	8.02	2.663-24.15	<0.001	5.39	1.329-21.86	0.02
Obesity	4.36	2.117-8.961	<0.001	2.96	1.201-7.312	0.02
Hypertension	1.74	0.878-3.446	0.11			
Hypertriglyceridemia	4.52	2.128-9.604	0.001	3.43	1.347-8.739	0.001
Nephropathy	10.7	4.765-24.19	<0.001	5.26	2.111-13.12	<0.001
Neuropathy	4.58	2.184-9.600	<0.001	4.52	1.734-11.80	0.002
Retinopathy	2.21	1.105-4.439	0.02			

GLS = global longitudinal strain; DM = diabetes mellitus; CI = confidence interval; OR = odds ratio.

### Association of clinical features with LV geometry and function

**Figure 3:**
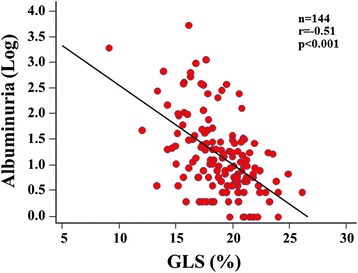
Dot plots of logarithmic albuminuria in relation to GLS show a significant negative correlation.

The findings obtained with the multiple linear regression analysis for association of clinical features with LV geometry and function is shown in Table 3. Albuminuria was the factor most closely associated with LV mass index (β = 0.27, p = 0.001) and GLS (β = −0.33, p < 0.001) even if adjusted for age, gender, DM duration, and systolic blood pressure. Albuminuria was also one of the independent determinative factors of E/E’ (β = 0.29, p = 0.001) together with age (β = 0.46, p < 0.001) and female gender (β = 0.22, p = 0.004). In addition, logarithmic albuminuria correlated negatively with GLS (r = −0.51, p < 0.001) (Figure 3). Interestingly, triglyceride correlated negatively with GLS (r = −0.41, p < 0.001), and was also one of the independent contributing factors of GLS (β = −0.24, p = 0.001), but low and high-density lipoprotein were not.

**Table 3:** **Association of clinical features with LV geometry and function**

**Variable**	**LV mass index**		**Relative wall thickness**		**LVEF**		**E/E’**		**GLS**	
	**β**	**p value**	**β**	**p value**	**β**	**p value**	**β**	**p value**	**β**	**p value**
Age					0.28	<0.001	0.46	<0.001		
DM duration										
Gender (female)					0.17	0.012	0.22	0.004		
Type 2 DM			0.28	<0.001						
Body mass index	0.18	0.023	0.29	<0.001					−0.20	0.006
Systolic blood pressure	0.22	0.007	0.21	0.005						
Triglyceride									−0.24	0.001
Albuminuria	0.27	0.001					0.29	0.001	−0.33	<0.001
Neuropathy			0.15	0.039					−0.18	0.014
Retinopathy			0.18	0.016	0.20	0.012				

Abbreviations as Table 1.

## Discussion

### Comparison to other studies of the prevalence of subclinical LV longitudinal systolic dysfunction in DM patients

This study confirms previous reports describing the subclinical LV longitudinal systolic dysfunction in DM patients. Nakai et al. reported that GLS in DM patients was significantly lower than that in age-matched normal subjects despite of similar LVEF, and 43% (26/60) of DM patients showed LV longitudinal systolic dysfunction determined as GLS < 17.2%[10]. In addition, Ernande et al. showed that 23% (36/154) of DM patients with preserved LVEF had LV longitudinal systolic dysfunction determined as GLS < 18%[13]. Our study showed 37% (53/144) of DM patients with preserved LVEF. Although the prevalence of subclinical LV longitudinal systolic dysfunction in DM patients with preserved LVEF varied among studies, this may depend on the patient characteristics such as the severity of DM or DM-related complications.

### Subclinical LV longitudinal systolic dysfunction in DM patients without overt HF

Pathophysiological causes of LV longitudinal dysfunction in DM patients are microvasculopathy, myocardial hypertrophy and cardiac fibrosis [1]. The transforming growth factor beta, aberrant differentiation of fibroblast progenitor cells due to hyperinsulinemia, and dysregulation of extracellular matrix due to hyperglycemia are also recognized as causes of not only renal but also cardiac fibrotic mechanism [27-29]. Ernande et al. prospectively studied 154 asymptomatic DM patients with preserved LVEF of ≥50% without overt heart disease to evaluate the association of LV longitudinal function with LV remodeling [13]. They reported that LV remodeling had progressed in patients with GLS < 18% at 3-year follow-up, but not in those with GLS ≥ 18%. In addition, GLS was independently associated with changes in both LV end-systolic and -diastolic volumes over the 3-year period. Indeed, diabetic cardiomyopathy is currently defined in terms of diastolic dysfunction, which is the earliest functional alteration in the course of diabetic cardiomyopathy [5-8]. However, LV diastolic dysfunction has been established as an important prognostic parameter [6], while LV diastolic function is affected by many other factors, such as age, hypertension, and LV hypertrophy. Ernande et al. also proved the presence of LV longitudinal dysfunction in DM patients with preserved LVEF of ≥55%, as assessed by GLS, despite these patients’ normal diastolic function. This indicates that diastolic dysfunction should not be considered the first marker of a preclinical form of diabetic cardiomyopathy [14].

### DM-related complications and LV longitudinal systolic myocardial dysfunction

Our study established that diabetic nephropathy and neuropathy were factors independently associated with LV longitudinal systolic myocardial dysfunction in asymptomatic DM patients with preserved LVEF. In addition, nephropathy had stronger influence on reduced GLS than neuropathy in multivariate logistic regression. Close cardio-renal connection can make us understood the dominance of nephropathy [30]. Therefore, some previous investigators have focused on the interaction of albuminuria and LV functions in DM patients [30-35]. Moreover, albuminuria has been viewed as a mirror of microvascular dysfunction, which results in vascular leaking of not only albumin, collagen and cholesterol, but also advanced glycation end products, and is the primary mediator of myocardial fibrosis due to the suppression of collagen turnover via impaired crosslinking of collagen [29,36]. Similarly, metabolic and vascular factors are involved in the pathophysiology of diabetic peripheral neuropathy. Metabolic factors include increased deposition of sorbitol, fructose, advanced glycation end products, and free oxygen radicals that are produced by uncontrolled hyperglycemia and cause painful damage to the peripheral nerve. It was also found that hyperglycemia generates microvascular ischemia, resulting in peripheral nerve damage due to vasoconstriction and microvasculopathy [37]. This phenomenon seems to be remarkably similar to the pathogenic mechanism of diabetic cardiomyopathy. On the other hand, among major DM-related complications only retinopathy was not an independent factor for the detection of GLS < 18%. A previous study demonstrated that deterioration of retinopathy is associated with LV diastolic function [38], but its association with LV longitudinal systolic function remains indeterminate. In fact, Karagöz et al. recently found no significant relationship between diabetic retinopathy and LV longitudinal systolic function in 82 asymptomatic patients with type 2 DM and preserved LVEF [39]. In this study, each retinopathy and neuropathy independently and mildly correlated with DM duration in multivariate model (r = 0.33, p = 0.001 and r = 0.19, p = 0.02), but nephropathy was not (p = 0.35). This finding indicates that evaluating exact uncontrolled-DM duration seems to be difficult and nephropathy may have a possible of closely association with cardiac function over DM duration which was obtained just from medical interview.

Overweight/obesity and hypertriglyceridemia were also identified as two of the contributing factors of GLS in our study, while other studies reported that obesity and metabolic syndrome were harmful factors for LV subclinical systolic and diastolic functioning [40-44]. Moreover, obesity is considered to be associated with hypertriglyceridemia, hyperinsulinemia, and insulin resistance [45-47]. Hypertriglyceridemia in particular is thought to be a cause of myocardial steatosis, resulting in subclinical LV systolic and diastolic dysfunction [48,49]. Our findings were thus agree with those of previous studies.

### Clinical implications

The pathogenesis of diabetic cardiomyopathy is considered to be multifactorial but the exact cause remains unknown. As previously stated, LV longitudinal systolic myocardial function could be a key parameter for the development of HF or LV remodeling in asymptomatic DM patients with preserved LVEF. However, what clinical features of DM patients are associated with impaired LV longitudinal systolic myocardial function has not been fully investigated. Our findings indicate that the highest risk factor of reduced GLS is diabetic nephropathy, and that albuminuria is the most closely associated with GLS. The detection of diabetic cardiomyopathy in the early stages is important for the prevention of HF that will develop in the future in asymptomatic DM patients with preserved LVEF. The new insights attained by our study therefore suggest that early detection of diabetic complications including diabetic nephropathy and neuropathy is important for the maintenance of LV longitudinal systolic function as well as the prevention of overweight for asymptomatic DM patients, even though their LVEF or LV diastolic function are preserved. It is therefore advisable for medical specialists, especially diabetologists, to jointly plan assessment for better management of DM patients.

### Study limitations

This cross-sectional study covered a relatively small number of patients in a single center study, so that future studies of larger patient populations with longitudinal cohort design are necessary to assess our findings. Our study populations included both type 1 (28%) and type 2 (72%) DM patients. However, when we performed the analyses for type 2 DM patients only, the overall results were similar. Finally, the confounding factors for logistic regression analysis and sequential logistic regression models were based on previously reported findings, so that a more complete description for this analysis may necessary.

## Conclusions

The assessment of diabetic complications, hypertriglyceridemia overweight/obesity could prove to be important for detecting of early stage of LV myocardial dysfunction in asymptomatic DM patients despite preserved LVEF. Our findings thus may well have clinical implications for better management of DM patients.

## Abbreviations

BMI: Body mass index
CI: Confidence interval
DM: Diabetes mellitus
E: Early diastolic wave velocity
E’: Spectral pulsed-wave Doppler-derived early diastolic velocity from the septal mitral annulus
EF: Ejection fraction
GFR: Glomerular filtration rate
GLS: Global longitudinal strain
HF: Heart failure
LAVI: Left atrial volume index
OR: Odds ratio
